# First Report of the Human-Pathogenic *Enterocytozoon bieneusi* from Red-Bellied Tree Squirrels (*Callosciurus erythraeus*) in Sichuan, China

**DOI:** 10.1371/journal.pone.0163605

**Published:** 2016-09-28

**Authors:** Lei Deng, Wei Li, Xingming Yu, Chao Gong, Xuehan Liu, Zhijun Zhong, Na Xie, Shuangshuang Lei, Jianqiu Yu, Hualin Fu, Hongwei Chen, Huailiang Xu, Yanchun Hu, Guangneng Peng

**Affiliations:** 1 The Key Laboratory of Animal Disease and Human Health of Sichuan Province, College of Veterinary Medicine, Sichuan Agricultural University, Chengdu, Sichuan, 611130, China; 2 The Chengdu Zoo, Institute of Wild Animals, Chengdu, Sichuan, 625001, China; 3 College of Life Science, Sichuan Agricultural University, Ya’an, Sichuan, 625014, China; Kent State University, UNITED STATES

## Abstract

*Enterocytozoon bieneusi* is a common opportunistic pathogen causing diarrhea and enteric disease in a variety of animal hosts. Although it has been reported in many animals, there is no published information available on the occurrence of *E*. *bieneusi* in red-bellied tree squirrels. To understand the occurrence, genetic diversity, and zoonotic potential of *E*. *bieneusi* in red-bellied tree squirrels, 144 fecal specimens from Sichuan province, China, were examined by PCR amplification and sequencing of the internal transcribed spacer (ITS) region of the ribosomal RNA (rRNA) gene of *E*. *bieneusi*. The overall infection rate of *E*. *bieneusi* 16.7% (24/144) was observed in red-bellied tree squirrels. Altogether five genotypes of *E*. *bieneusi* were identified: three known genotypes D (n = 18), EbpC (n = 3), SC02 (n = 1) and two novel genotypes CE01, CE02 (one each). Multilocus sequence typing (MLST) analysis employing three microsatellite (MS1, MS3, MS7) and one minisatellite (MS4) revealed 16, 14, 7 and 14 positive specimens were successfully sequenced, and identified eight, three, three and two genotypes at four loci, respectively. In phylogenetic analysis, the three known genotypes D, EbpC, and SC02 were clustered into group 1 with zoonotic potential, and the two novel genotypes CE01 and CE02 were clustered into group 6. The present study firstly reported the occurrence of *E*. *bieneusi* in red-bellied tree squirrels in China, and the *E*. *bieneusi* genotypes D and EbpC were found in humans previously. These results indicate that red-bellied tree squirrels may play a potential role in the transmission of *E*. *bieneusi* to humans.

## Introduction

Microsporidia, obligate intracellular eukaryotic pathogens, are composed of approximately 1300 species in 160 genera [1, 2]. Currently, at least 14 microsporidia species in eight genera have been detected in humans [3]. *Enterocytozoon bieneusi* is the most prevalent microsporidian species and accounts for more than 90% of the cases of human microsporidiosis [4, 5]. Generally, infective spores of *E*. *bieneusi* are excreted through feces of infected animals into the environment, and are capable of infecting susceptible humans, especially children, via consumption of contaminated food and water [3]. In humans, clinical symptoms caused by *E*. *bieneusi* in immunocompetent individuals are self-limiting diarrhea and malabsorption. Most seriously, *E*. *bieneusi* cause a life-threatening diarrhea in immune-compromised patients, particularly in AIDS patients and organ transplant recipients [3, 6]. Apart from humans, *E*. *bieneusi* has been observed in many vertebrates species, including mammals, reptiles, and birds [7–10].

Sequence analysis of the internal transcribed spacer (ITS) region of the ribosomal RNA (rRNA) gene is the standard method for genotyping *E*. *bieneusi* due to a high degree of gene tic polymorphism within *E*. *bieneusi* isolates in humans and animals [4, 9]. Thus far, molecular epidemiological surveys of *E*. *bieneusi* in different parts of the world demonstrate over 240 genotypes in animals and humans [11–16]. All the published genotypes of *E*. *bieneusi* have been divided into eight different groups via the phylogenetic analysis [17]. Group 1, considered as the human pathogenic group, contains almost all the *E*. *bieneusi* genotypes from humans and some genotypes from animals [18]. In contrast, the remaining clusters that form the groups 2 to 8 are mostly found in specific hosts and wastewater [5, 19]. However, the use of single ITS maker may be inadequate in identifying genotypes of *E*. *bieneusi* due to the uncertainty about whether meiotic recombination occurs in *E*. *bieneusi* lifecycle [15]. Recently, a multilocus sequencing typing (MLST) analysis employing three microsatellites (MS1, MS3, MS7) and one minisatellite (MS4) makers has been developed to better know the route of transmission, genetic diversity and host specificity of *E*. *bieneusi* [15, 20, 21].

In China, *E*. *bieneusi* has been confirmed in humans, animals, and water samples [13, 16, 17, 22–26], but only limited reports about rodents are available, and their role as reservoirs of infection for humans and other animals are still unknown. The red-bellied tree squirrels, as commercial and companion animals, are known to be closely associated with humans. In recent years, they have gained more popularity among various groups of people, especially children. Nevertheless, there has been no research conducted on the infection rates and genetic characterization of *E*. *bieneusi* in red-bellied tree squirrels. To the best of our knowledge, the current study is the first to explore the occurrence and genetic diversity of *E*. *bieneusi* in red-bellied tree squirrels, and to evaluate the zoonotic potential in transmission of human microsporidiosis.

## Materials and Methods

### Ethics statement

This study was conducted in accordance with the Guide for the Care and Use of Laboratory Animals of the Ministry of Health, China. The research protocol was reviewed and approved by the Research Ethics Committee of the Sichuan Agricultural University, Sichuan, China. Permission was obtained from the animal owners or managers before collecting the fecal specimens. During the collection of fecal specimens, the animals were not subjected to any kind of injury.

### Collection of specimens

From March 2014 to September 2015, a total of 144 fecal specimens were collected from pet owners, pet shops, and breeding facility in Ya'an, Nanchong, and Chengdu, of Sichuan province, southwestern China (Table 1) (S1 Table). We used sterile disposable latex gloves during the collection of each fresh specimen. The samples were placed into individual 30 ml plastic containers, and then were transported to the laboratory with ice packs within 24 h of collection. At the same time, data on the source, age, gender, and physical condition of each animal were recorded. The red-bellied tree squirrels were grouped according to their age as follows: <3 months (n = 55), 3 to 12 months (n = 67), and >12 months (n = 22) (Table 2). None of these experimental animals presented with any diarrheic or gastrointestinal conditions.

**Table 1:** Occurrence and genotypes of *E*. *bieneusi* in red-bellied tree squirrels from different cities and sources of southwest China.

City	Source	No. of animals	No. of positive (%)	Genotypes (n)
Ya'an	Pet shop1	35	7(20.0)	D(3), EbpC(2), CE01(1), SC02(1)
Nanchong	Pet shop2	23	4(17.4)	D(4)
Chengdu	Pet shop3	12	2(16.7)	D(1), EbpC(1)
	Owner	25	3(12.0)	D(3)
	Breeding facility	49	8(16.3)	D(7), CE02(1)
Total		144	24(16.7)	D(18), EbpC(3), SC02(1) CE01(1), CE02(1),

**Table 2:** Occurrence and genotypes of *E*. *bieneusi* in red-bellied tree squirrels by age and gender.

Group	No. of animals	No. of positive (%)	Genotypes(n)
Age(month)			
<3	55	11(20.0)	D(8), EbpC(2), SC02(1)
3–12	67	9(13.4)	D(7), CE01(1), CE02(1)
>12	22	4(18.2)	D(3), EbpC(1)
Gender			
Male	61	10(16.4)	D(8), CE01(1), SC02(1)
Female	83	14(16.9)	D(10), EbpC(3), CE02(1)

### DNA extraction

Each fecal specimen was sieved, and the filtrates were concentrated and washed three times with distilled water by centrifugation for 10 min at 1500 g. Genomic DNA was extracted from approximately 200 mg of each processed fecal specimen using the E.Z.N.A.® Stool DNA Kit (D4015–02; OMEGA Biotek Inc., Norcross, GA, USA) according to the manufacturer’s instructions. The extracted DNA was stored at -20°C until PCR analysis.

### PCR amplification

All the DNA preparations were examined for the presence of *E*. *bieneusi* by nested PCR amplification of a fragment of 389 bp in size from the rRNA gene of *E*. *bieneusi*, and positive specimens were further determined by MLST analyses using the MS1, MS3, MS4, and MS7 loci. The primers and cycling parameters employed for these reactions were as previously described (Table 3) [20, 27]. TaKaRa Taq DNA Polymerase (TaKaRa Bio Inc., Tokyo, Japan) was used for the PCR amplifications. A negative control with no DNA was set up for all the PCR tests. Secondary PCR products were subjected to electrophoresis in a 1.5% agarose gel and visualized by staining the gel with ethidium bromide.

**Table 3:** Gene locus, primer sequences, annealing temperatures and fragment length for the identification of *E*. *bieuensi* used in this study.

Gene locus	Primer sequence (5'-3')	Annealing temperature (°C)	Fragment length (bp)	References
ITS	F1:GATGGTCATAGGGATGAAGAGCTT	55	410	[27]
	R1:AATACAGGATCACTTGGATCCGT			
	F2:AGGGATGAAGAGCTTCGGCTCTG	55	392	
	R2:AATATCCCTAATACAGGATCACT			
MS1	F1: CAAGTTGCAAGTTCAGTGTTTGAA	58	843	[20]
	R1: GATGAATATGCATCCATTGATGTT			
	F2:TTGTAAATCGACCAAATGTGCTAT	58	676	
	R2:GGACATAAACCACTAATTAATGTAAC			
MS3	F1:CAAGCACTGTGGTTACTGTT	55	702	[20]
	R1:AAGTTA GGGCATTTAATAAAATTA			
	F2:GTTCAAGTAATTGATACCAGTCT	55	537	
	R2:CTCATTGAATCTAAATGTGTATAA			
MS4	F1:GCATATCGTCTCATAGGAACA	55	965	[20]
	R1:GTTCATGGTTATTAATTCCAGAA			
	F2:CGA AGTGTACTACATGTCTCT	55	885	
	R2: GGACTTTAATAAGTTACCTATAGT			
MS7	F1:GTTGATCGTCCAGATGGAATT	55	684	[20]
	R1:GACTATCAGTATTACTGATTATAT			
	F2:CAATAGTAAAGGAAGATGGTCA	55	471	
	R2:CGTCGCTTTGTTTCATAATCTT			

### Nucleotide sequencing and analysis

The secondary PCR products of the expected size were directly sequenced at Life Technologies (Guangzhou, China) using an ABI Big Dye Terminator v3.1 cycle sequencing kit (Applied Biosystems, Carlsbad, CA, USA). The accuracy of the sequences was confirmed by bidirectional sequencing, and a new PCR secondary product was re-sequenced, if necessary.

The sequences generated in this study were respectively aligned with known reference sequences downloaded from the National Center for Biotechnology Information (NCBI) GenBank database by using BLAST (http://www.ncbi.nlm.nih.gov) to determine their genotype identity. The genotypes, identified as identical to the known genotypes, were assigned the already published names. Meanwhile, the genotypes with single nucleotide substitutions, deletions or insertions compared to the known genotypes were considered novel genotypes, and then named according to the established nomenclature system [9].

### Phylogenetic relationship of *E*. *bieneusi*

To assess the genetic relationship of ITS genotypes of *E*. *bieneusi* obtained in the present study and those published in the previous studies, a phylogenetic analysis was performed by constructing a neighboring-joining tree using the software Mega 6 (http://www.megasoftware.net/), based on the evolutionary distances calculated by a Kimura 2-parameter model. The reliability of these trees was assessed using bootstrap analysis with 1000 replicates.

### Statistical analysis

The χ^2^ test was used to compare the *E*. *bieneusi* infection rates between the sex, age and different sampling areas, and differences were considered significant when p < 0.05.

### Nucleotide sequence accession numbers

Representative nucleotide sequences have been deposited into GenBank database with the following accession numbers: KU847350 to KU847351 for the rRNA gene ITS sequences of two novel genotypes obtained in the present study (CE01, CE02), and KX259505 to KX259519 for the microsatellite loci (MS1, MS3, MS7) and minisatellite (MS4).

## Results

### Occurrence of *E*. *bieneusi* in red-bellied tree squirrels

Among the 144 fecal samples, 24 were positive for *E*. *bieneusi* (16.7%) by PCR amplification of the ITS gene. The infection rates of *E*. *bieneusi* in different sources ranged from 12.0% (3/25) in owners to 20.0% (7/35) in pet shop 1 (Table 1), but the difference were not found to be significant (P>0.05). Infection rates of *E*. *bieneusi* in red-bellied tree squirrels of different ages and genders have been presented in Table 2; the highest infection rate was observed in <3 months (20.0%, 11/55), followed by 18.2% (4/22) in >12 months, and 13.4% (9/67) in 3–12 months of age (with non-significant differences, P>0.05). Male and female red-bellied tree squirrels showed an infection rate of 16.4% (10/61) and 16.9% (14/83), respectively; however, the difference was not significant (P>0.05).

### Genotype distribution and genetic characterizations of *E*. *bieneusi* in red-bellied tree squirrels

DNA sequencing and subsequent analysis of the ITS-PCR products from the 24 *E*. *bieneusi*-positive specimens revealed the existence of three known *E*. *bieneusi* genotypes (D, EbpC, SC02), and two novel genotypes, which were named as CE01 and CE02 (Table 1). Genotype D was the most prevalent (75.0%, 18/24), and was observed in samples from all the three cities, followed by EbpC, which was detected in three specimens from Chengdu and Ya'an cities (12.5%, 3/24); and the genotypes, SC02, CE01, and CE02 were found in one specimen each collected from Ya'an and Chengdu (4.7%, 1/24) (Table 1).

With regard to the novel genotypes, CE01 displayed five single nucleotide polymorphisms (SNPs) within the 243 bp of the ITS gene sequence of *E*. *bieneusi* (transversions: T/G, G/T; transitions: A/G, C/T, G/A), when compared to the genotype horse 2 (KU194600), with 99% homology; CE02 had one SNP (transition: C/T) in comparison with genotype horse 2, with 99% homology.

### Phylogenetic relationship of *E*. *bieneusi*

**Fig 1:**
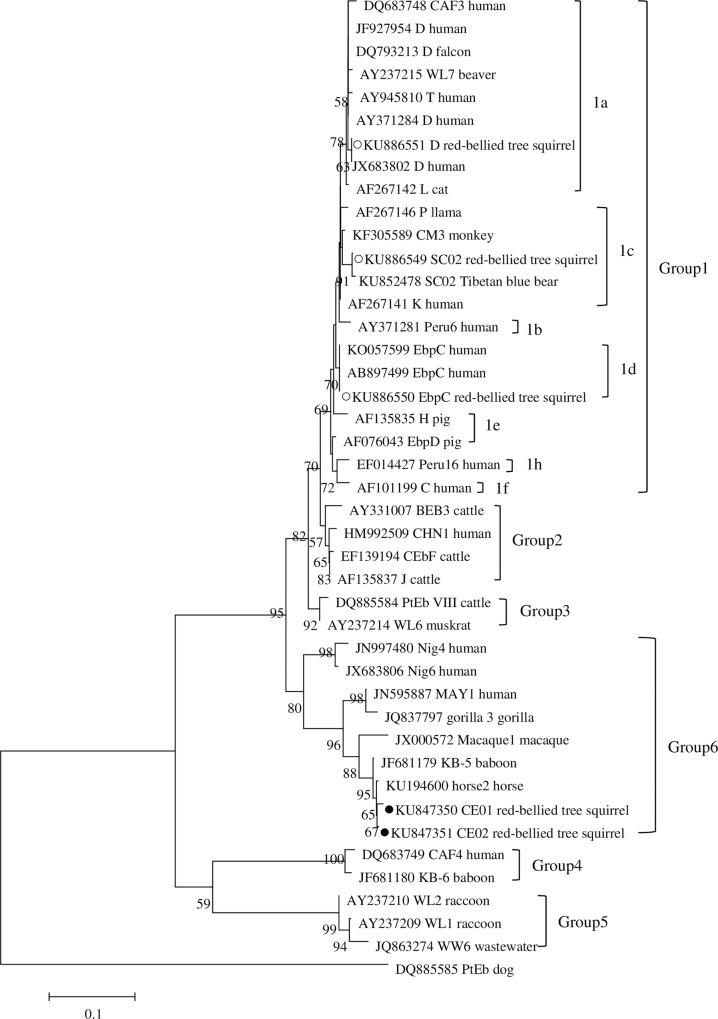
Phylogenetic relationship of *Enterocytozoon bieneusi* groups, the relationship between *E*. *bieneusi* genotypes identified in this study and other known genotypes deposited in the GenBank was inferred by a neighbor-joining analysis of ITS sequences based on genetic distance by the Kimura-2-parameter model. The numbers on the branches represent percent bootstrapping values from 1,000 replicates, with more than 50% shown in tree. Each sequence is identified by its accession number, genotype designation, and host origin. The group terminology for the clusters is based on the work of Zhao et al [28]. Genotypes with *black circles* and *open circles* are novel and known genotypes identified in this study, respectively.

A phylogenetic analysis using neighbor-joining method based on the ITS gene sequences of *E*. *bieneusi* showed that all positive samples found in the present study belonged to two groups. Genotypes D, EbpC, and SC02 were clustered into group 1 and were further classed into subgroup 1a, 1d, and 1c, respectively (Fig 1). The two novel genotypes, CE01 and CE02, were clustered into group 6 (Fig 1).

### Multilocus sequence typing of *E*. *bieneusi*

ITS-positive specimens were further characterized using one minisatellite (MS4) locus and three microsatellites (MS1, MS3 and MS7) loci. A total of 16, 14, 7 and 14 fecal samples were successfully amplified at the MS1, MS3, MS4, and MS7 loci, respectively, and then sequencing analysis revealed 8, 3, 3 and 2 genotypes at the MS1, MS3, MS4, and MS7 loci, respectively. Only 6 samples were simultaneously amplified and sequenced at four loci, sequencing analysis formed five distinct MLGs, namely MLG1-5 (Table 4). All MLGs (MLG1-5) were observed in genotypes D (Table 4).

**Table 4:** Multilocus characterization of *E*. *bieneusi* isolates from red-bellied tree squirrels in Sichuan, southwestern China.

ITS genotype	Multilocus genotypes					MLGs	No. of MLGs
	MS1	MS3	MS4	MS7	GenBank accession Nos.		
D	Type3	Type1	Type2	Type2	KX259510, KX259513, KX259516, KX259519	MLG1	1
D	Type3	Type1	Type2	Type1	KX259510, KX259513, KX259516, KX259518	MLG2	1
D	Type1	Type1	Type2	Type1	KX259508, KX259513, KX259516, KX259518	MLG3	2
D	Type8*	Type2*	Type2	Type1	KX259509, KX259514, KX259516, KX259518	MLG4	1
D	Type7*	Type2*	Type3	Type1	KX259511, KX259514, KX259517, KX259518	MLG5	1

*Novel genotypes

## Discussion

*E*. *bieneusi* is an emerging zoonotic pathogen and has been reported in humans as well as many animals, such as cattle, pigs, dogs, cats, horses, goats, birds, giant pandas, red pandas, deer, snakes, and golden takins [6, 16, 18, 23, 26, 28–30]. To our knowledge, the present study is the first to reveal the presence of *E*. *bieneusi* in red-bellied tree squirrels in China, with an infection rate of 16.7% (24/144). Although the rate of infection of *E*. *bieneusi* in red-bellied tree squirrels was frequent in <3 months (20.0%), no age-associated differences were observed in this study. This finding, which was in accordance with a previously published report on chinchillas in China [31], may be attributed to the fact that young animals have incomplete immune system and are prone to the intensive breeding environments. Despite considerable research on this pathogen, only a few genetic studies have documented the occurrence of *E*. *bieneusi* in rodents (Table 5); for instance, the highest infection rate of *E*. *bieneusi* was observed in wild small rodents (38.9%) in Poland [32], followed by 26.8% in wild rodents from New York [11], 15.3% in beavers in Maryland [27], 10.7% in wild mice from Czech Republic [33], 3.6% in chinchillas in China [31], and 1.0% in wild mice from Slovakia [34]. The observed infection rate was lower than that reported in Poland and New York City, and it was higher than that estimated in Czech Republic, China, and Slovakia.

**Table 5:** Distribution of *E*. *bieneusi* genotypes in red-bellied tree squirrels from different countries.

Country	Host	No. positive/no. examined (%)	Genotypes	Reference
Poland	Wild rodents	121/311 (38.9%)	D, gorilla 1, WR1-WR10	[31]
United States	Wild rodents	38/142 (26.8%)	Peru11, Type IV, WL4, WW6, PtEbV, WL20, WL21, WL22, WL23, WL25	[11]
United States	Beavers	13/85 (15.3%)	WL7, WL8, WL9, WL12, WL13, WL15	[27]
Czech Republic	Wild mice	31/289 (10.7%)	D, EpbA, PigEBITS5, C, H, CZ3, Peru 8, S6	[32]
China	Chinchillas	5/140 (3.6%)	D, BEB6	[33]
Slovakia	Wild mice	3/280 (1.0%)	Peru16	[34]

Analysis of the ITS region of the ribosomal RNA revealed a total of five distinct genotypes out of the 24 *E*. *bieneusi* isolates, which comprised of three known genotypes (D, EbpC, and SC02) and two novel genotypes (CE01 and CE02). The genotype D showed the highest percentage of *E*. *bieneusi*-positive specimens in the present study, accounting for 75.0% (18/24), followed by genotype EbpC (12.5%; 3/24) (Table 1). In China, genotype D has an extensive host range and has been examined in humans [35], non-human primate, pigs, dogs, foxes, and cats [13, 36–39], as well as in waste water [22]. Besides, the genotype EbpC has been previously observed in many animals, including humans, cattle, pigs, sheep, dogs, non-human primates, deer, beavers, otters, muskrats, raccoons, and foxes, even giant panda [23, 24, 40]. Interestingly, the two genotypes, D and EbpC, which were examined for the first time in red-bellied tree squirrels in the current study, presented an expanded host range. These results indicated that red-bellied tree squirrels may play a potential role in the transmission of *E*. *bieneusi* to humans.

Genetic relationship of two novel genotypes of *E*. *bieneusi* to the known ones was observed in a phylogenetical analysis. The two novel genotypes (CE01 and CE02) were clustered into group 6. Genotypes WW7, WW8 were first detected in urban wastewater belonging to group 6 [22] and then recent studies have revealed that certain genotypes from other animals were also clustered into group 6, including gorilla 3 in gorillas, KB-5, and Macaque1 in non-human primates, Horse 2 in horses [33, 41, 42]. Meanwhile, other members of this group, such as genotypes Nig4, Nig6, MAY1, have also been reported in humans [10, 35, 43, 44]. Therefore, it is not completely known whether the two novel genotypes belonging to group 6 have an ability to cause microsporidiosis in humans, and the potential of zoonotic transmission need to be confirmed by future extensive genotyping research in large samples of human microsporidiosis.

In order to better understand route of transmission, genetic diversity and host specificity of *E*. *bieneusi*, the MLST tool for subtyping *E*. *bieneusi* was developed [20]. In the present study, sequencing analysis indicated 8, 3, 3 and 2 genotypes at the MS1, MS3, MS4, and MS7 loci, respectively, and identified three, one, one novel genotypes in loci MS1, MS3, MS4, respectively. A total of five distinct MLGs (MLG1-5) were observed in genotypes D. These results showed the genetic diversity of *E*. *bieneusi* in red-bellied tree squirrels.

In conclusion, this is the first report on the occurrence of three known human-pathogenic *E*. *bieneusi* genotypes (D, EbpC, SC02) and two novel genotypes (CE01, CE02) in red-bellied tree squirrels in Sichuan province, China. Genetic diversity was observed by MLST tool, and five MLGs were found in red-bellied tree squirrels. The fact that genotypes D and EbpC have been previously reported in humans, suggest that red-bellied tree squirrels can serve as potential reservoir hosts for the zoonotic transmission of human microsporidiosis. Due to the high frequency of human contact with pet animals in China, proper advice should be given to the susceptible human populations in order to reduce the zoonotic transmission of this neglected disease.

## Supporting Information

S1 Table Basic information of red-bellied tree squirrels from different cities. (XLSX)
