# Correction: First Report of the Human-Pathogenic *Enterocytozoon bieneusi* from Red-Bellied Tree Squirrels (*Callosciurus erythraeus*) in Sichuan, China

**DOI:** 10.1371/journal.pone.0168631

**Published:** 2016-12-19

**Authors:** Lei Deng, Wei Li, Xingming Yu, Chao Gong, Xuehan Liu, Zhijun Zhong, Na Xie, Shuangshuang Lei, Jianqiu Yu, Hualin Fu, Hongwei Chen, Huailiang Xu, Yanchun Hu, Guangneng Peng

The first four authors, Lei Deng, Wei Li, Xingming Yu and Chao Gong, should be noted as contributing equally to this work.
